# Behavior of Neutrophil Granulocytes during *Toxoplasma gondii* Infection in the Central Nervous System

**DOI:** 10.3389/fcimb.2017.00259

**Published:** 2017-06-21

**Authors:** Aindrila Biswas, Timothy French, Henning P. Düsedau, Nancy Mueller, Monika Riek-Burchardt, Anne Dudeck, Ute Bank, Thomas Schüler, Ildiko Rita Dunay

**Affiliations:** ^1^Institute of Inflammation and Neurodegeneration, Otto-von-Guericke University MagdeburgMagdeburg, Germany; ^2^Institute for Molecular and Clinical Immunology, Otto-von-Guericke University MagdeburgMagdeburg, Germany

**Keywords:** *Toxoplasma gondii*, neutrophil granulocytes, cerebral toxoplasmosis, neuroinflammation, neutrophil Infiltration

## Abstract

Cerebral toxoplasmosis is characterized by activation of brain resident cells and recruitment of specific immune cell subsets from the periphery to the central nervous system (CNS). Our studies revealed that the rapidly invaded Ly6G^+^ neutrophil granulocytes are an early non-lymphoid source of interferon-gamma (IFN-γ), the cytokine known to be the major mediator of host resistance to *Toxoplasma gondii* (*T. gondii*). Upon selective depletion of Ly6G^+^ neutrophils, we detected reduced IFN-γ production and increased parasite burden in the CNS. Ablation of Ly6G^+^ cells resulted in diminished recruitment of Ly6C^hi^ monocytes into the CNS, indicating a pronounced interplay. Additionally, we identified infiltrated Ly6G^+^ neutrophils to be a heterogeneous population. The Ly6G^+^CD62-L^hi^CXCR4^+^ subset released cathelicidin-related antimicrobial peptide (CRAMP), which can promote monocyte dynamics. On the other hand, the Ly6G^+^CD62-L^lo^CXCR4^+^ subset produced IFN-γ to establish early inflammatory response. Collectively, our findings revealed that the recruited Ly6G^+^CXCR4^+^ neutrophil granulocytes display a heterogeneity in the CNS with a repertoire of effector functions crucial in parasite control and immune regulation upon experimental cerebral toxoplasmosis.

## Introduction

*T. gondii* is a highly successful parasite capable of crossing most biological barriers of the body (Barragan, 2002; Harker et al., 2015). The parasite can infect migratory immune cells such as dendritic cells (DCs), macrophages and neutrophil granulocytes to enter immunoprivileged sites (Barragan, 2002; Da Gama et al., 2004; Courret et al., 2006; Lambert et al., 2006). During the acute phase of infection, enterocytes secrete chemokines including monocyte chemotactic protein-1 (MCP-1) and macrophage inflammatory protein-2 (MIP-2), which recruit neutrophil granulocytes, inflammatory monocytes and DCs to the site of infection (Robben et al., 2005; Courret et al., 2006; Pittman and Knoll, 2015). We have previously detected that the CD8α^+^ DC subset is the crucial source of interleukin-12 (IL-12), the cytokine that stimulates natural killer (NK) cells and T cells to produce the cytokine IFN-γ (Mashayekhi et al., 2011). IFN-γ is a major mediator of host resistance to *T. gondii*, regulating a plethora of intracellular mechanisms to eliminate the parasite (Gazzinelli et al., 1993; Hunter et al., 1994; Kang and Suzuki, 2001; Kim and Weiss, 2004; Gazzinelli and Sher, 2014). Recent studies revealed that neutrophil granulocytes, besides innate lymphoid cells, NK cells and T cells, are able to produce IFN-γ (Sturge et al., 2013). They also produce IFN-γ independently from toll-like receptor (TLR)11-induced IL-12 during acute toxoplasmosis in the periphery (Sturge et al., 2013, 2015). Our previous experiments provided evidence that unlike inflammatory monocytes, neutrophil granulocytes worsened ileal pathology upon specific depletion in the acute phase of toxoplasmosis (Dunay et al., 2010). These studies were carried out in mice, which had an intact TLR-recognition system responsible for IL-12 dependent IFN-γ production by NK and T cells. However, the relative contribution of neutrophil granulocytes to IFN-γ production was not elucidated.

The parasites can enter the CNS within immune cells, initially infecting endothelial cells of the blood-brain barrier, where they egress, and invade brain resident cells (Konradt et al., 2016). Cyst formation within neurons undergoing a stress-mediated response is followed by ongoing basal inflammation, establishing a chronic persistent infection (Parlog et al., 2014; Blanchard et al., 2015). Chronic cerebral toxoplasmosis is characterized by the activation of resident cells such as microglia and astrocytes, which carry out distinct antiparasitic functions (Strack et al., 2002; Schluter et al., 2003; Drögemüller et al., 2008; Kamerkar and Davis, 2012; Cabral et al., 2016). In addition, peripheral immune cell subsets of myeloid and lymphoid origin are recruited to the inflamed CNS (John et al., 2011). Recently, we characterized the behavior of infiltrating CD11b^+^Ly6C^hi^ myeloid derived cells, which carry out a pivotal role upon cerebral toxoplasmosis (Möhle et al., 2014, 2016; Biswas et al., 2015). Several studies have highlighted the role of brain-resident cells, lymphocytes, DCs, and macrophages (Strack et al., 2002; John et al., 2011), however, the function of neutrophil granulocytes in host defense in the CNS is still uncertain.

Neutrophil granulocytes differentiating from the common myeloid progenitor in the bone marrow (BM) form the first line of defense during infection and inflammation (Soehnlein et al., 2008b). Upon reaching the site of infection, neutrophils can execute many immune functions including production of reactive oxygen species (ROS) or proinflammatory modulators (Denkers et al., 2012; Bardoel et al., 2014; Perez-de-Puig et al., 2015). The surface marker Ly6G, exclusively expressed on neutrophil granulocytes, facilitates distinction from monocytes, enabling specific functional studies. The role of neutrophil granulocytes in the periphery and the CNS has been investigated in multiple neurodegenerative diseases and infections, however, their impact on cerebral toxoplasmosis remains to be elucidated (Zhou et al., 2003; Zehntner et al., 2005; Mildner et al., 2008; Ransohoff and Engelhardt, 2012; Sturge et al., 2013; Wantha et al., 2013; Marais et al., 2014).

Here we characterized the phenotype and behavior of Ly6G^+^ neutrophil granulocytes in chronic *T. gondii* infection. We detected their influx into the CNS during the acute stage of cerebral toxoplasmosis, which was associated with the upregulation of expression of certain activation markers and co-stimulatory molecules. Importantly, intracellular cytokine analysis revealed that neutrophil granulocytes form a non-lymphoid source of the pro-inflammatory cytokine IFN-γ at the acute stage of infection. Upon using a specific anti-granulocyte monoclonal antibody (mAb) 1A8, we measured a significant increase in the parasite burden. We also detected a reduction in monocyte recruitment and IFN-γ production confirming the specific contribution of neutrophil granulocytes. Neutrophils exhibited heterogeneity based on specific expression of the adhesion molecule CD62-L and the chemokine receptor CXCR4, respectively. This implies that multiple signals at the site of the inflammation can influence the functional characteristics and phenotype of neutrophils.

## Materials and methods

### Animals

Age and sex matched C57BL/6 wild-type (WT) mice, obtained from Janvier (Cedex, France) were used. All animal care was in accordance with institutional guidelines. Food and water were available *ad libitum*. Experiments were performed in accordance to German and European legislation.

### Infection

*T. gondii* cysts of type II strain ME 49 were harvested from the brains of female NMRI mice infected intra-peritoneally (i.p.) with *T. gondii* cysts 8–10 months earlier. Brains obtained from infected mice were mechanically homogenized in 1 ml sterile phosphate-buffered saline (PBS). Cyst numbers were counted in a 10 μl brain suspension using a light microscope. Two cysts were administered i.p. in a total volume of 200 μl per mouse as described before (Möhle et al., 2016). Control mice were mock-infected with sterile PBS. The mice were perfused intracardially with 60 ml sterile ice-cold PBS. The brains were removed for further analysis. The acute stage of infection was defined between day 10 and day 14 whereas the chronic stage of infection was starting at day 21. Therefore, to investigate the recruited neutrophil granulocytes in chronic cerebral toxoplasmosis, mice were sacrificed 4 weeks post-infection for further analysis.

### 1A8 mAb treatment

Depletion of neutrophils was performed by i.p. administration of anti-Ly6G mAb (clone 1A8, BioXCell). Mice were injected with 500 μg (as described before by Daley et al., 2008; Dunay et al., 2010) of the antibody i.p. on alternate days from days 10 to 23 post-infection. Mice were sacrificed 24 h after the last antibody treatment. Rat IgG2a (BioXCell) was used as a control to mAb.

### Cell isolation

Brains were homogenized in a buffer containing 1M HEPES (pH 7.3) and 45% glucose and then sieved through a 70 μm strainer. The cell suspension was washed and fractionated on 25–75% Percoll gradient (GE Healthcare). Isolated cells were washed with PBS and used immediately for further experiments. Peripheral blood was obtained from posterior vena cava and lysed with erythrocyte (RBC) lysis buffer (eBioscience). Subsequently, these cells were stained with the desired fluorescent conjugated antibodies (Biswas et al., 2015).

### Flow cytometry

Isolated mononuclear cells were incubated with Zombie NIR™ or Violet™ fixable dye (Biolegend) for live/dead discrimination. Unspecific antibody binding was blocked by incubation with anti-FcγIII/II receptor antibody (clone 93). Thereafter, cells were stained with fluorochrome-conjugated antibodies against cell surface markers: CD45 (30-F11), CD11b (M1/70), Ly6C (HK1.4), MHC I H-2D^b^ (28-14-8), MHC II I-A/I-E (M5/114.15.2), CD80 (16-10A1), and CD86 (GL1), all purchased from eBioscience, CXCR2 (SA04E1), CXCR4 (L27GF12), and CD64 (X54-5/7.1) from Biolegend, Ly6G (1A8), CD62-L (MEL-14) from BD Bioscience, then washed and fixed in 4% paraformaldehyde. Matched isotype controls were used to assess the level of unspecific binding.

For intracellular cytokine staining, single-cell suspensions (5 × 10^5^ cells/well) were stimulated in 96-well plates in the presence of *Toxoplasma* lysate antigen (5 μg/ml) and Brefeldin A (10 μg/ml, GolgiPlug, BD Biosciences). After 6 h, cells were incubated with Zombie NIR™ or Violet™ fixable dye and anti-FcγIII/II receptor antibody (clone 93) and surface stained for CD45 (30-F11), CD11b (M1/70), Ly6G (1A8), Ly6C (HK1.4), CRAMP (R-170), washed in FACS buffer [PBS with 1% of fetal calf serum (FCS)] and fixed in 4% paraformaldehyde. Cells were permeabilized using Permeabilization Buffer (Biolegend). To measure the cytokine expression, the staining was performed with the following antibodies: IL-1α (ALF-161), TNF (MP6-XT22), NOS2 (CXNFT), IFN-γ (XMG1.2), IL-1β (NJTEN3), and IL-10 (JES5-16E3) from eBioscience in Permeabilization Buffer (Biolegend) (Biswas et al., 2015).

A total of 100,000 cells was acquired using a flow cytometer (BD FACS Canto II). Data were analyzed using FlowJo software (Version 10 Tree Star). Matched isotype controls were used to assess the level of unspecific binding.

### Detection of reactive oxygen species

Isolated cells were stained for CD45 (30-F11), CD11b (M1/70), Ly6G (1A8), Ly6C (HK1.4), CXCR4 (L27GF12), and CD62-L (MEL-14) in FACS buffer for 30 min after blocking FcγRs. ROS production was measured by Total ROS Detection Kit (ENZO, 51011), according to the manufacturer's instructions.

### q- and qRT-PCR

RNA and DNA was isolated from the right hemisphere of the infected mice as previously described (Möhle et al., 2016). Following the isolation of the nucleic acids, semi-quantitative PCR was performed to measure the parasite burden and the cytokine gene expression levels in the brain.

### Immunofluorescence

Mice were anesthetized with isoflurane and perfused intracardially with saline followed by paraformaldehyde (PFA, 4%) in phosphate buffer (pH 7.4). The brain was removed, post-fixed with PFA overnight, cryoprotected in 30% sucrose, frozen, and 20-μm-thick sections were prepared in a cryostat. The sections were fixed with ethanol, blocked with normal serum and incubated overnight at 4°C with the primary antibody SAG-1 (D61S clone; 1:20, Invitrogen) or PECAM-1/CD31 (MEC13.3 clone; 1:500) and Ly6G (1A8 clone; 1:200). Following the primary antibody staining, lectin staining was performed (Fluorescein labeled Dolichos Biflorus Agglutinin (DBA); 1:500, Vector Laboratories) according to the manufacturer's guidelines. Then, sections were incubated for 2 h at room temperature with secondary antibodies (Alexa Fluor-488, Thermo Fischer Scientific). Controls were carried out by omission of the primary antibodies. Sections were counterstained with Sytox Red Dead Cell Stain (1:20,000, Thermo Fischer Scientific) to visualize the cell nuclei and slides were observed under a confocal laser microscope (LSM510, Carl Zeiss).

### Statistical analysis

Data were analyzed by Mann-Whitney test for two groups or one-way ANOVA for several groups followed by Tukey's post-test with GraphPad Prism 6 (San Diego, CA). In all cases, results were presented as mean ± standard deviation (SD) and were considered significant, with *p* < 0.05.

## Results

### Rapid influx of neutrophil granulocytes into the brain upon cerebral toxoplasmosis

**Figure 1:**
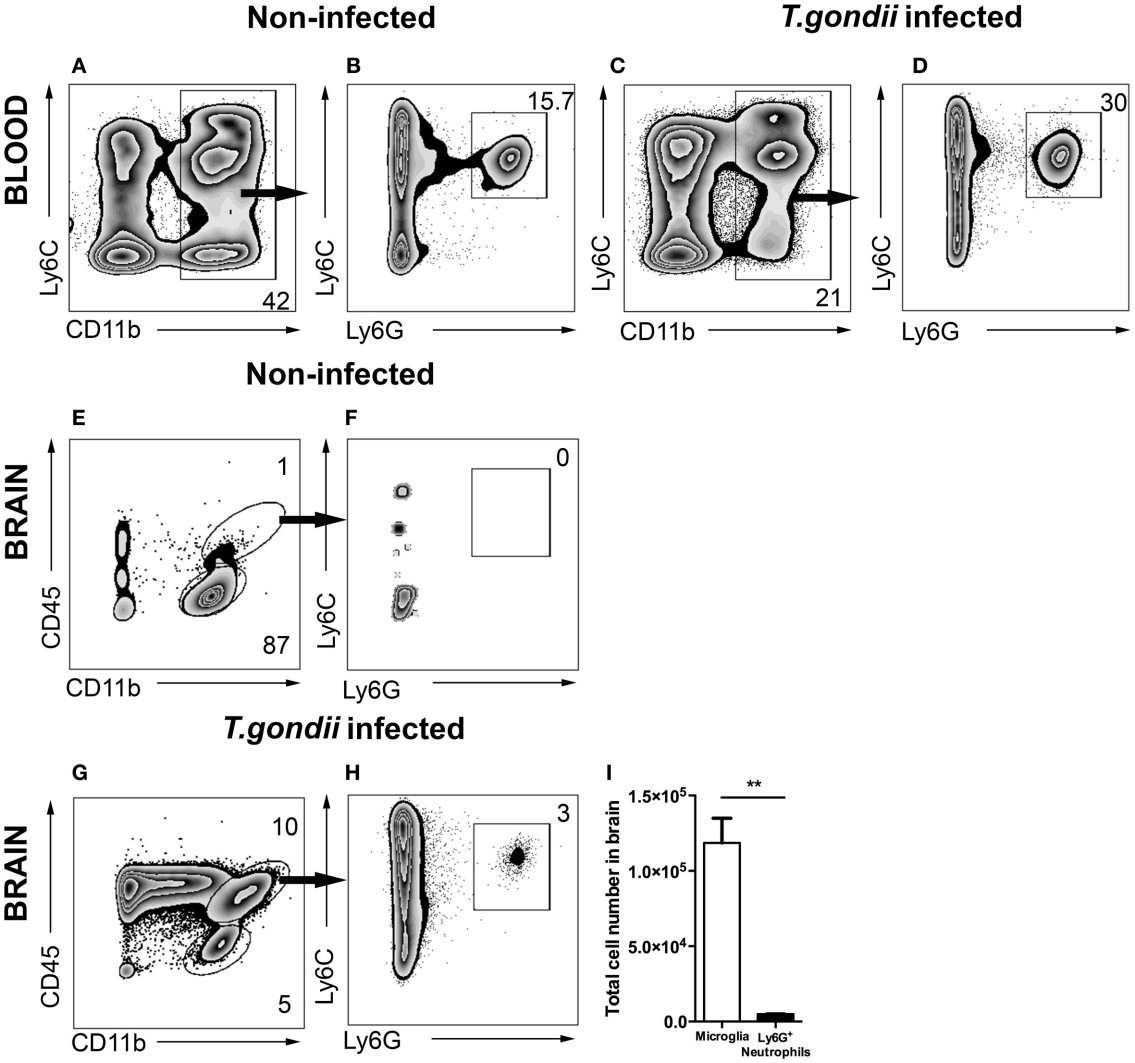
Gating strategy of neutrophil granulocytes in blood and in brain. Cells were isolated from the blood and brain of non-infected mice **(A,B,E,F)** and the blood and brain of 4 weeks *T. gondii*-infected mice **(C,D,G,H)** and analyzed by flow cytometry. Following FSC-SSC, singlet gating and exclusion of dead cells (not shown), live cells were used for further analysis. **(A,C)** Show gating of CD11b^+^ cells in the blood which are further characterized into Ly6C^+^Ly6G^+^ neutrophil granulocytes **(B,D)**. **(E)** Shows the resident microglia (CD11b^+^CD45^lo^, lower gate), and **(F)** shows the percentage of neutrophil granulocytes (Ly6C^+^Ly6G^+^) from myeloid populations in the brains of non-infected animals (CD11b^+^CD45^hi^, upper gate). **(G,H)** Shows the exact same gating strategy in the infected brains. **(I)** The bar graphs represent total number of cells (activated microglia - white bar and neutrophil granulocytes - black bar) in the infected brains. Numbers represent % of parent population. The data shown is representative for 3 independent experiments with 5 mice per group for each experiment. Mann-Whitney test was performed for comparisons (^**^p < 0.01).

Infection of mice with *T. gondii* induces egress of Ly6C^hi^ monocytes and Ly6G^+^ neutrophil granulocytes from the BM to the blood. Following our previous characterization of BM derived monocytes in the CNS in 4 weeks *T. gondii*-infected mice, we analyzed neutrophil dynamics using the same experimental model. CD11b and Ly6C expression were used to identify the inflammatory monocytes (Ly6C^hi^), neutrophils (Ly6C^int^) and resident monocytes (Ly6C^lo^) in the blood (Figures 1A,C). The CD11b^+^ cells were further differentiated based on their Ly6C and Ly6G expression (Figures 1B,D). The neutrophil specific Ab Ly6G (1A8) was applied to distinguish neutrophil granulocytes (Ly6G^+^) from monocytes (Ly6G^−^) (Figures 1B,D). We observed increased percentages of circulating neutrophil granulocytes in peripheral blood of infected mice (30 ± 3.06%) as compared to non-infected controls (15.7 ± 1%; Figures 1B,D).

Alongside the enhanced egress of immune cells in the periphery, cerebral toxoplasmosis leads to the activation of brain resident cells and infiltration of circulating immune cells to the CNS (Möhle et al., 2014; Biswas et al., 2015). Peripheral immune cell influx into the infected brain is characterized by initial invasion of neutrophils, followed by the recruitment of BM-derived monocytes and the lymphocytes. While in non-infected controls the main immune cell population was resting resident microglia (CD45^lo^CD11b^+^; Figures 1E,F), we observed the entry of CD45^hi^ myeloid derived population in the brains of *T. gondii*-infected mice (Figure 1G). The recruited cells included two populations, CD45^hi^CD11b^−^ cells (ungated; Figure 1G) and CD45^hi^CD11b^+^ cells (upper gate; 10.0 ± 2.1% of the parent population). The CD45^hi^CD11b^+^ cells comprised of BM-derived myeloid cells, namely monocytes, neutrophil granulocytes, macrophages and DCs. Upon infection, brain resident activated microglia cells expressed elevated CD45 levels (CD45^int^CD11b^+^; 5.0 ± 1.06% of the parent population). The anti-Ly6G (1A8) antibody was applied to distinguish Ly6G^+^ neutrophil granulocytes (Figure 1H; 3.0 ± 1.02% of the CD45^hi^CD11b^+^) from monocytes. Next, the total number of mononuclear cells was quantified in the brain during chronic cerebral toxoplasmosis (Figure 1I). Thus, CD45^hi^CD11b^+^Ly6G^+^ neutrophil granulocytes (6 × 10^3^ ± 50) formed a small yet defined population, when compared to the absolute numbers of CD45^int^CD11b^+^ microglia (1 × 10^5^ ± 2,000). These data demonstrate that cerebral toxoplasmosis leads to the activation of resident microglia and the recruitment of lymphoid and myeloid immune cells including neutrophil granulocytes.

### Localization of neutrophil granulocytes in the CNS during the course of cerebral toxoplasmosis

**Figure 2:**
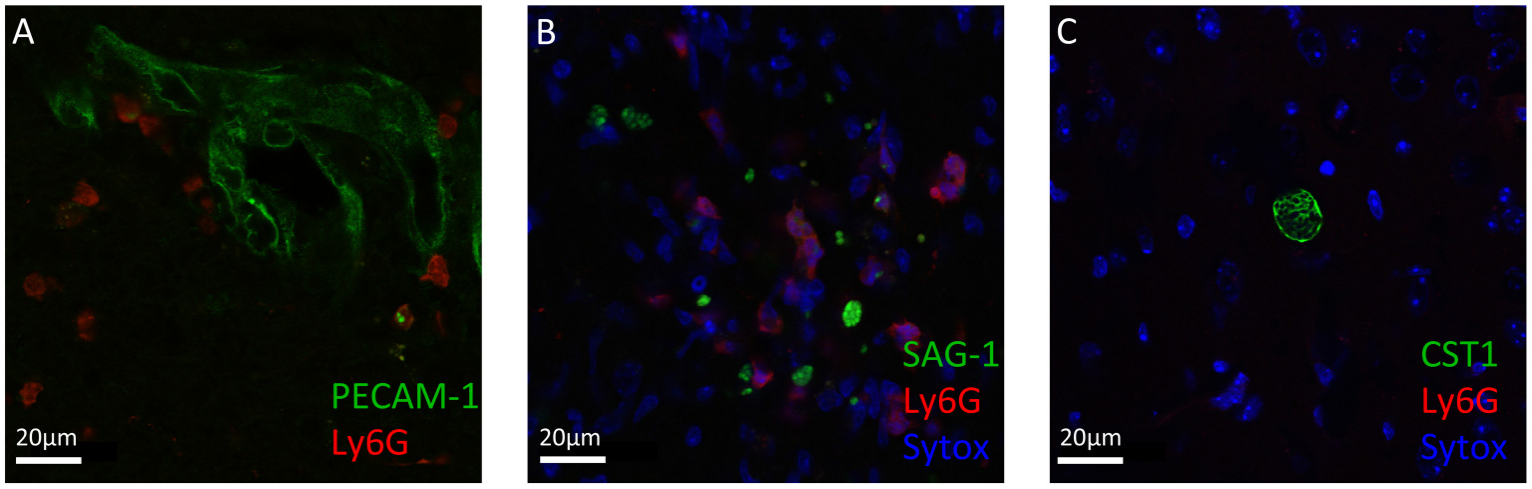
Immunofluorescence staining of Ly6G^+^ neutrophil granulocytes and *T. gondii* in brain slices. Immunostaining of PECAM-1 or SAG1 or CST1 (green), Ly6G (red), and Sytox Dead Cell stain (blue) in the cortex of *T. gondii*-infected C57BL/6 mice **(A–C)**. **(A)** In acute cerebral toxoplasmosis, recruitment of Ly6G^+^ neutrophil granulocytes into the brain from the circulation is shown. **(B)** Further, massive infiltration of Ly6G^+^ neutrophil granulocytes around *T. gondii* tachyzoites (green) is shown. **(C)** In chronic cerebral toxoplasmosis, no localization of Ly6G^+^ neutrophil granulocytes is shown. Eight coronal slides per mouse were analyzed with a maginification of 63x; *n* = 4 mice. This experiment was repeated two times. Scale bars, 20 μm in **(A–C)**.

Neutrophil granulocytes, the earliest immune cells to arrive at the site of infection, can deploy a plethora of mechanisms to attack invading pathogens (Denkers et al., 2012; Bardoel et al., 2014; Perez-de-Puig et al., 2015). To study their localization around *T. gondii* in the CNS, we performed immunofluorescence analysis of mice brain slices at the acute stage and chronic stage of the infection. We observed that neutrophil granulocytes were not only in the blood vessels, marked by PECAM1, but entered the brain parenchyma (Figure 2A). The recruited neutrophils were in close proximity to the *T. gondii* tachyzoites in the cerebral cortex during the acute stage infection (Figure 2B). Upon encystation of the *T. gondii* parasites in the chronic phase, we observed the absence of neutrophils in the vicinity of bradyzoite containing cysts (Figure 2C). These immunofluorescence results indicate the affinity of the neutrophil granulocytes to the infective stage of *T. gondii* in contrast to the dormant stage during the course of the cerebral toxoplasmosis.

### Phenotypic analysis of infiltrating neutrophils upon cerebral *T. gondii* infection

**Figure 3:**
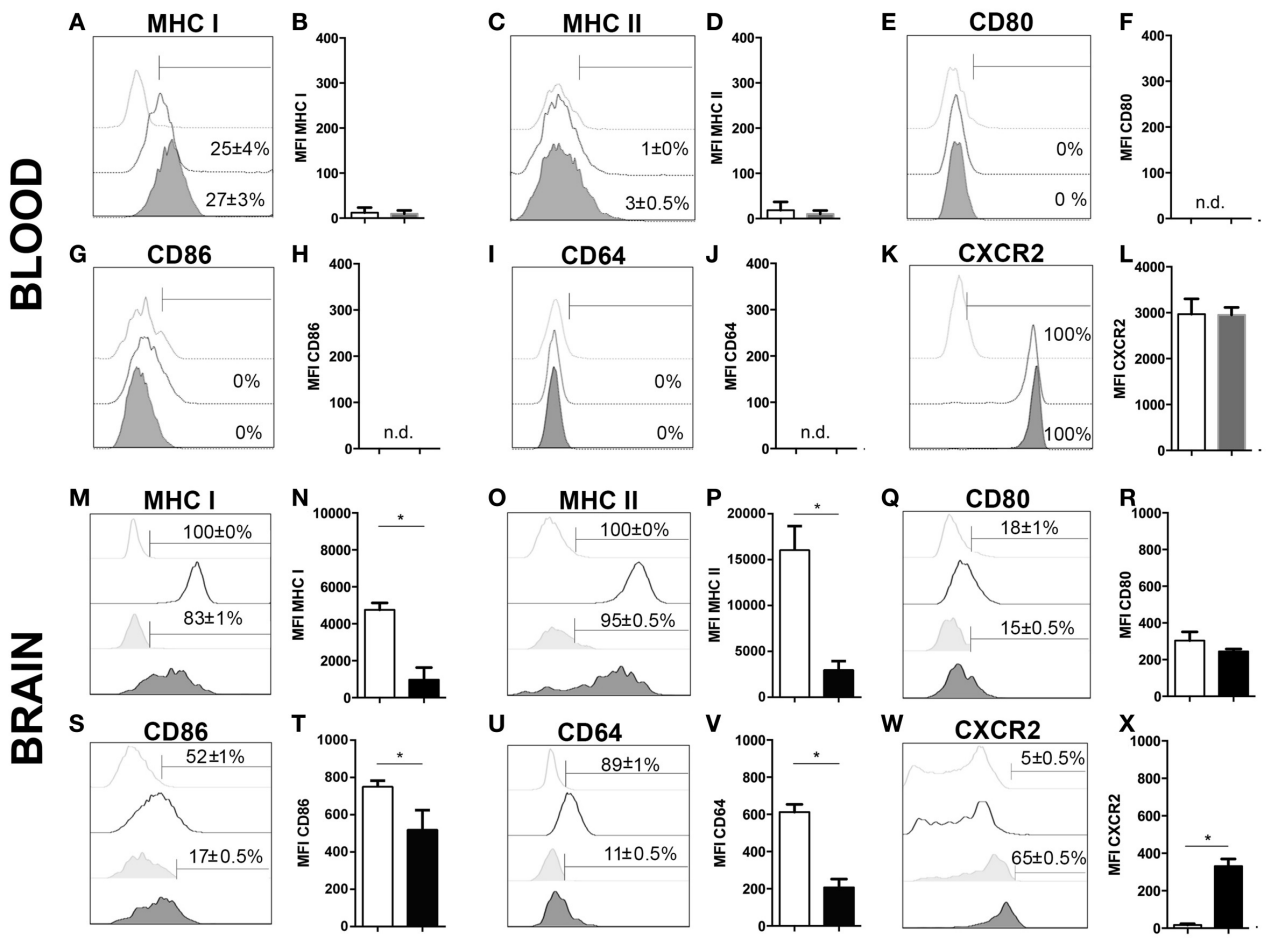
Phenotypic analysis of neutrophil granulocytes and activated microglia **(A–X)**. Expression of activation markers and chemokine receptors in mice blood and brains after 4 weeks of *T. gondii* infection, were analyzed by flow cytometry, respectively. The cells were gated as described and shown in the representative plots of Figure 1. Neutrophils from the infected and non-infected blood (CD11b^+^CD45^hi^Ly6G^+^Ly6C^+^), activated microglia (CD11b^+^CD45^int^), and neutrophil granulocytes from the infected brain (CD11b^+^CD45^hi^Ly6G^+^Ly6C^+^), were assessed for their relative expression of the indicated molecules. **(A,C,E,G,I,K,M,O,Q,S,U,W)** Histograms show the representative expression level of the surface maker by the cell population in comparison to the corresponding isotype control (light gray without tint or light gray tinted). Bars mark cells positively expressing particular surface markers and numbers above bars represent the percentage of cells in the respective population: neutrophils in non-infected blood (CD11b^+^Ly6G^+^) (without any tint, dotted line), neutrophils in infected blood (CD11b^+^Ly6G^+^) (tinted, dotted line), activated microglia (CD11b^+^CD45^int^) (without any tint), neutrophil granulocytes (CD11b^+^Ly6G^+^) (tinted). **(B,D,F,H,J,L,N,P,R,T,V,X)** Bar graphs represent the median fluorescence intensity (MFI) for the specific marker MFI ± SD (*n* = 4) (neutrophils in non-infected blood: white bars; neutrophils in infected blood: gray bars; activated microglia: white bars; and neutrophils: black bars). Data represent 2 independent experiments with 5 mice per experiment. Mann-Whitney test was performed for comparisons (^*^p < 0.05).

To further characterize neutrophil granulocytes in inflamed brains upon 4 weeks of *T. gondii* infection, the expression profiles of various cell surface markers were determined by flow cytometry. As control, microglia were selected, because under homeostatic condition neutrophils are not present in the CNS, and immune cells from the same isolate chosen for the comparison. A large percentage of infiltrating neutrophils expressed enhanced levels of MHC I and MHC II (83 ± 1% MHC I; 95 ± 0.5% MHC II) (Figures 3M–P) unlike in the periphery (25 ± 4% MHC I; 3 ± 0.5% MHC II) (Figures 3A–D). According to their function as antigen presenting cells (APCs) of the brain, activated microglia also expressed significant levels of MHC I and MHC II (100% MHC I; 100% MHC II) (Figures 3M–P). We further compared the expression of the co-stimulatory molecules CD80 and CD86 and detected these on small parts of the neutrophil population (15 ± 0.5% CD80; 17 ± 0.5% CD86) and activated microglia (18 ± 1% CD80; 52 ± 1% CD86) (Figures 3Q–T). The phagocytosis mediating receptor CD64 (FcgR1), which was present on a large proportion of activated microglia (89 ± 1% CD64), was only detectable on a small percentage of neutrophils (11 ± 0.5%) (Figures 3U,V). The chemokine receptor CXCR2 was detected on more than half of the neutrophils (65 ± 0.5%). However, only a small percentage of activated microglia expressed CXCR2 (5 ± 0.5%) (Figures 3W,X). The neutrophil granulocytes in the periphery did not express any significant levels of CD80, CD86 and CD64 (Figures 3E–J). However, the entire neutrophil population expressed CXCR2 (Figures 3K,L). Hence, activated microglia and neutrophils express immune modulatory molecules in infected brains indicating their contribution to the establishment of local immunity in response to *T. gondii* infection.

### Cytokine production of infiltrating neutrophils upon cerebral *T. gondii* infection

**Figure 4:**
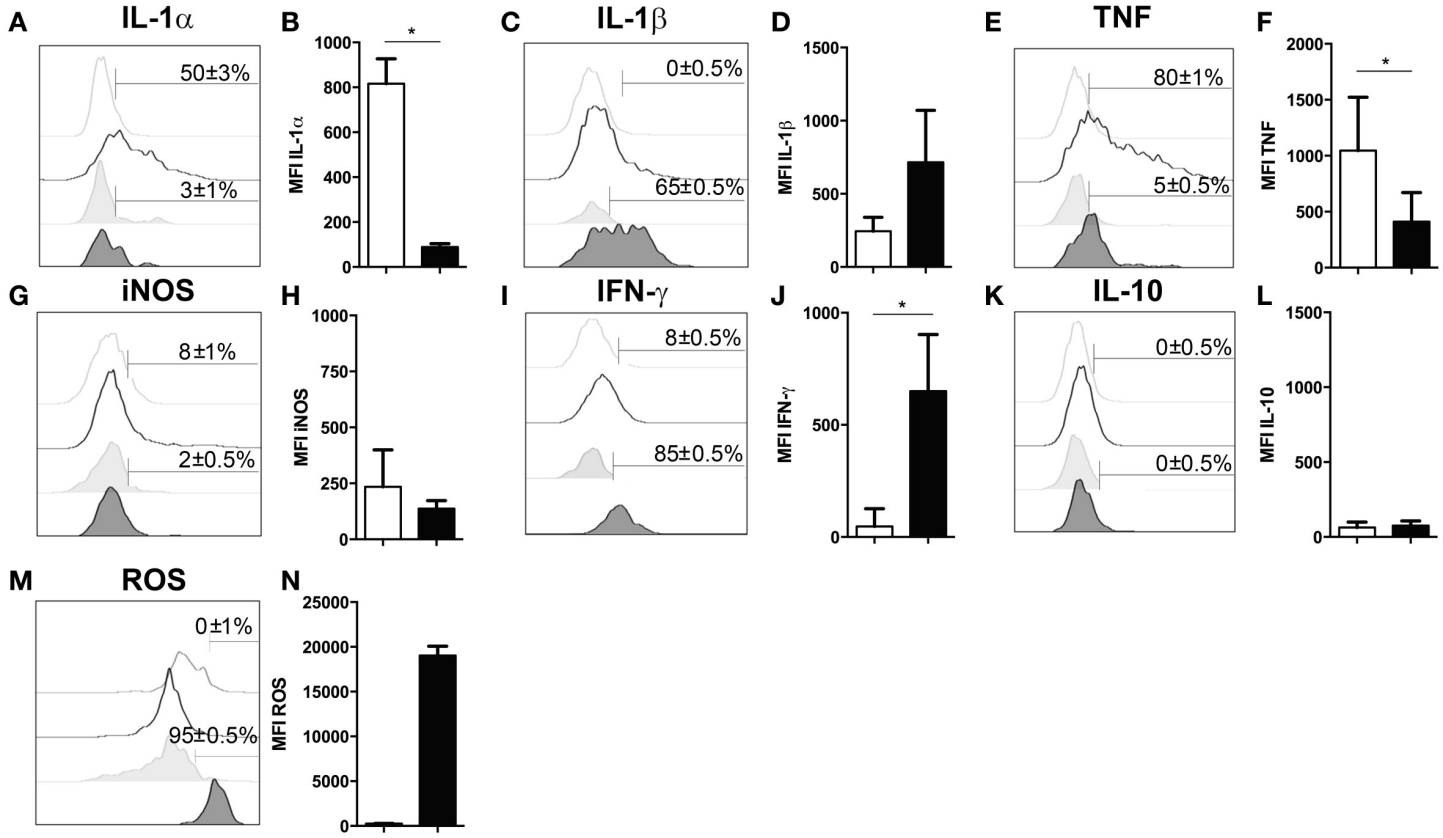
Functional properties of neutrophil granulocytes and activated microglia in the infected brain. **(A–N)** Cells isolated from brains of mice infected with 4 weeks of *T. gondii*, were re-stimulated with *Toxoplasma* lysate antigen *in vitro* and analyzed by flow cytometry. The cells were gated as described in the Figure 1 **(E–H)**. **(A,C,E,G,I,K,M)** Histograms show the representative expression levels of the indicated molecules in comparison to the corresponding isotype control (light gray without tint or light gray tinted). Bars mark cells positive for the particular cytokine. Numbers above bars represent the percentage of cells positive for the cytokine of the respective population: activated microglia (CD11b^+^CD45^int^) (without any tint), neutrophil granulocytes (CD11b^+^Ly6G^+^) (tinted). **(B,D,F,H,J,L,N)** Bar graphs represent the MFI of the respective fluorochrome for a particular cytokine, MFI ± SD (activated microglia: white bars; and neutrophils: black bars). Data are representative of 2 independent experiments with 5 mice per experiment. Mann-Whitney test was performed for comparisons (^*^p < 0.05). White bars and black bars represent activated microglia and neutrophil granulocytes respectively.

Several studies have demonstrated that neutrophil granulocytes secrete plenty of immune modulatory molecules including cytokines upon infection (Sturge et al., 2013; Bardoel et al., 2014). To determine their cytokine expression profile, in experimental cerebral toxoplasmosis (4 weeks *T. gondii* infection), myeloid cells from the infected brain were analyzed by intracellular flow cytometry. We observed that neutrophil granulocytes hardly produced pro-inflammatory cytokines such as IL-1α and TNF (3 ± 1% and 5 ± 0.5%, respectively). On the contrary, microglia served as a considerable source of both cytokines (50 ± 3% and 80 ± 1%, respectively) (Figures 4A,B,E,F). Importantly, 65 ± 0.5% of neutrophil granulocytes synthesized IL-1β (Figures 4C,D). Interestingly, IFN-γ was also produced by the neutrophils (85 ± 0.5%) contrary to activated microglia (Figures 4I,J). IL-10 and iNOS were synthesized by negligible numbers of neutrophil granulocytes (0 ± 0.5% IL-10; 2 ± 0.5% iNOS) and microglial cells (0 ± 0.5% IL-10; 8 ± 1% iNOS) (Figures 4G,H,K,L). Besides, all neutrophils synthesized ROS unlike activated microglia (95 ± 0.5%) (Figures 4M,N). In summary, these data demonstrate that neutrophil granulocytes produce multiple immune modulatory molecules including IFN-γ.

### IFN-γ production by neutrophil granulocytes over the course of cerebral toxoplasmosis

**Figure 5:**
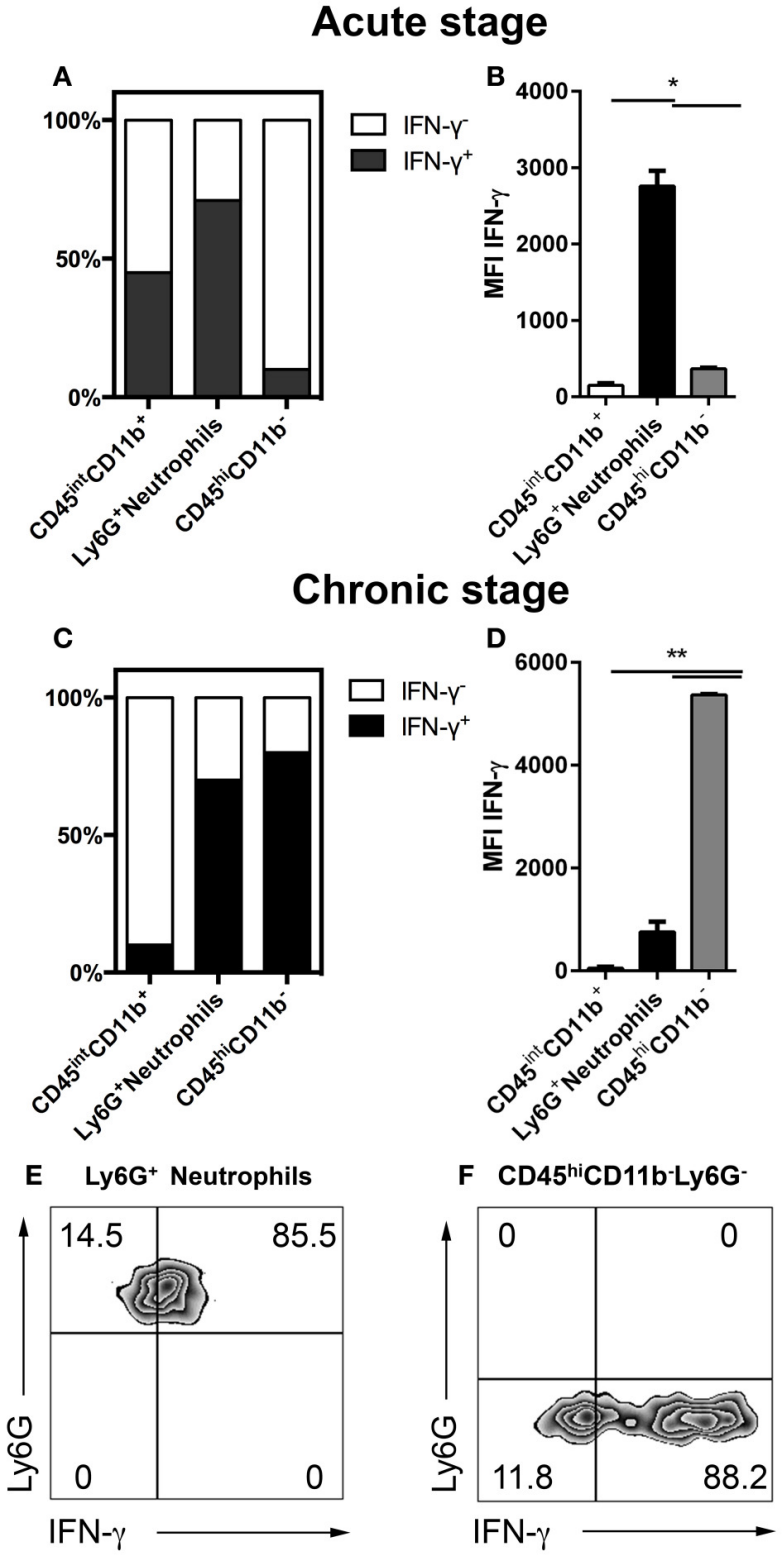
IFN-γ production over the course of cerebral toxoplasmosis **(A–F)**. Cells isolated from brains of mice infected with 2 and 4 weeks of *T. gondii*, were re-stimulated with *Toxoplasma* lysate antigen *in vitro* and analyzed by flow cytometry. The cells were gated as shown in Figures 1E,F. **(A,C)** The fraction of the total cell population expressing IFN-γ was plotted in stacked bar graphs. **(B,D)** Bar graphs represent the MFI of the respective fluorochrome for a particular cytokine, MFI ± SD (*n* = 4). **(E,F)** The representative plots show the gating strategy of Ly6G^+^ neutrophils and CD11b^−^CD45^hi^Ly6G^−^ cells producing IFN-γ. The quadrant was set on isotype control. Data are representative of 2 independent experiments with 5 mice per experiment. Significant differences (^*^p < 0.05, ^**^p < 0.01) were determined using the Mann-Whitney test. White bars, black bars and gray bars represent CD45^int^CD11b^+^ activated microglia, Ly6G^+^ neutrophils and CD45^hi^CD11b^−^ lymphocytes, respectively.

IFN-γ plays a critical role in the host response to cerebral toxoplasmosis. Previous studies defined NK cells, T cells and microglia as sources of IFN-γ during cerebral toxoplasmosis (Gazzinelli et al., 1993; Hunter et al., 1994; Gavrilescu et al., 2004; Kim et al., 2012; Gazzinelli and Sher, 2014). However, the contribution of neutrophil granulocytes to IFN-γ production in the brain has not been elucidated (Suzuki, 2002; Sa et al., 2015). Therefore, we performed flow cytometry from the brains of mice during the acute (2 weeks) as well as the chronic stage (4 weeks) of *T. gondii* infection. In the acute phase, 83.5 ± 0.5% of neutrophil granulocytes produced IFN-γ (1.5 ^*^ 10 ^∧^ 3 ± 200 cells). This was the case for only 47 ± 0.5% of activated microglia. At this time-point, only negligible numbers of CD11b^−^ lymphocytes (247 ± 50 cells) were detectable in the brain to make a significant contribution (6 ± 0.5%) (Figures 5A,B). However, during the chronic phase of infection enhanced number of lymphocytes infiltrated the brain, which then became the major IFN-γ producers (88 ± 0.5%) (1.5 ^*^ 10 ^∧^ 6 ± 550 cells) (Figures 5C–F). Neutrophils were secondary producers of IFN-γ at the chronic stage of infection (85 ± 4%) (1.4 ^*^ 10 ^∧^ 3 ± 60 cells) (Figures 5C–F). Thus, our results demonstrate that Ly6G^+^ neutrophil granulocytes are representing an important early source of early IFN-γ in the acute phase of cerebral toxoplasmosis.

### Depletion of neutrophil granulocytes in experimental cerebral toxoplasmosis

**Figure 6:**
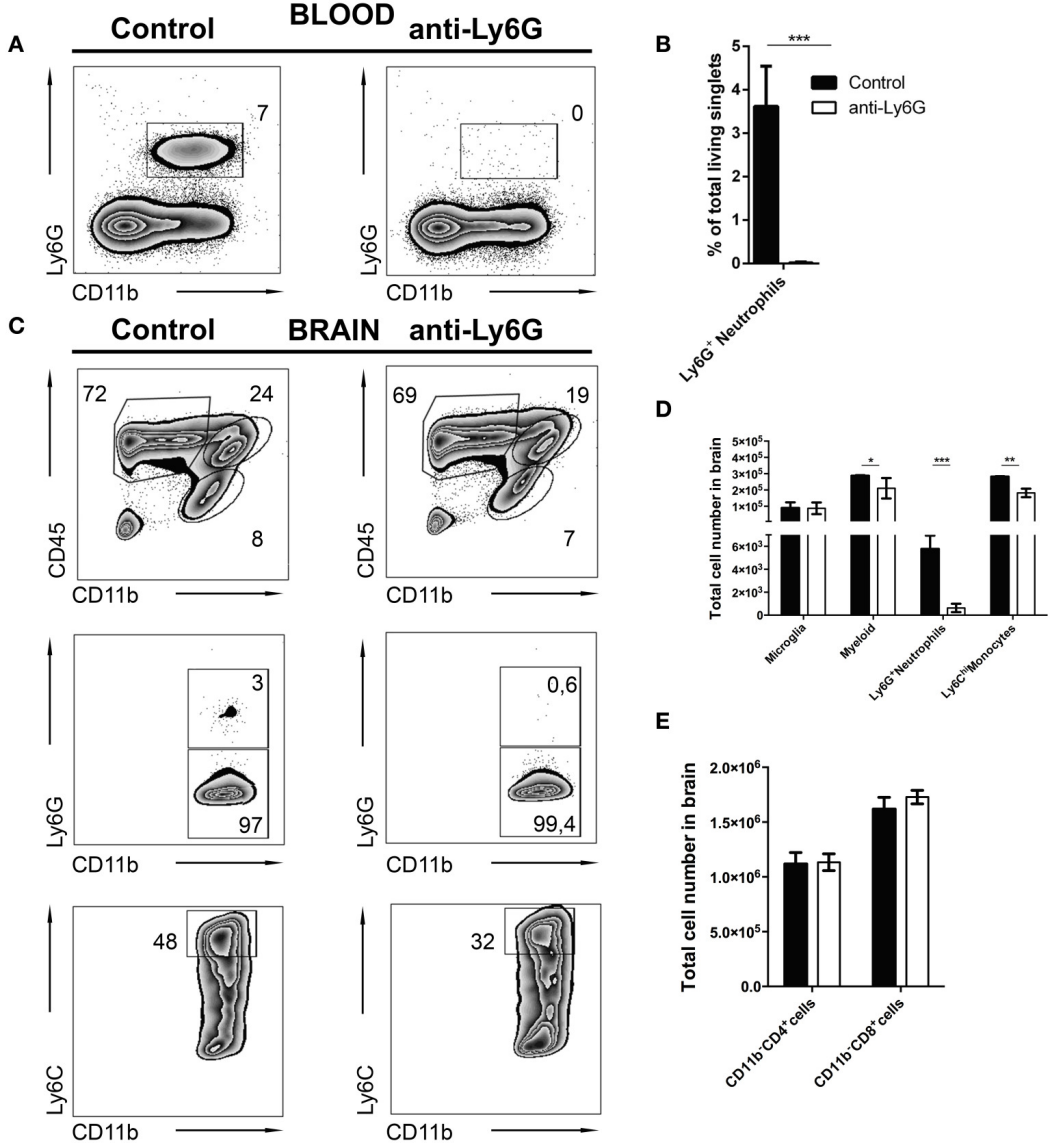
Selective depletion of Ly6G^+^ neutrophil granulocytes. C57BL/6 mice were infected with *T. gondii*. From day 10 to day 23 post-infection mice were alternatively treated with either IgG mAb (control, left), or anti-Ly6G (Anti-Ly6G, right) to deplete neutrophil granulocytes. The cells were gated as shown in Figures 1E–H. Panel **(A)** displays representative plots to define CD11b^+^Ly6G^+^ circulating neutrophil granulocytes (upper gate) in the blood. **(B)** The bar graph represents the percentage of Ly6G^+^ cells in the blood. **(C)** The upper plots show the gating of lymphocytes (CD11b^−^CD45^hi^), activated microglia (CD11b^+^CD45^int^) and the myeloid population (CD11b^+^CD45^hi^) in infected brains. Middle plots display the myeloid subsets: Ly6G^+^(neutrophil granulocytes) and Ly6G^−^ (monocytes) in the brain. Lower plots display the inflammatory monocyte subset (from the Ly6G^−^ gate): Ly6C^hi^. **(D)** The bar graphs represent the total cell number of the respective subsets in the brain (black bars: control; white bars: anti-Ly6G). **(E)** The bar graphs represent the total cell number of the respective CD4^+^ and CD8^+^ lymphoid cell population. Data shown here is the representative of 2 individual experiments with 4 mice per group for each experiment. The numbers in the representative contour plots are % of the parent population. Significant differences (^*^p < 0.05, ^**^p < 0.01, ^***^p < 0.001) were determined using the Mann-Whitney test.

It is still unclear whether neutrophils contribute to control of cerebral toxoplasmosis. To investigate this, we took advantage of the previously described specific depleting anti-Ly6G mAb (clone 1A8). Following our observation that Ly6G^+^ neutrophils are early IFN-γ producers in the acute phase of cerebral toxoplasmosis, we started the ablation at day 10 post-infection when neutrophil granulocytes began entering the brain. We continued the ablation, injecting Ly6G mAb or control IgG2 mAb every alternate day, as the infection progressed from the acute to chronic stage. Twenty-four hours after the last Ab treatment, mice were sacrificed and the successful depletion of CD11b^+^ Ly6G^+^ monocytes in the blood was confirmed (7.0 ± 0.13 to 0%; Figures 6A,B).

In agreement with this, anti-Ly6G treatment of infected mice also resulted in a significant reduction of neutrophils in the brain. Further characterization revealed a reduction of recruited myeloid cells (24.0 ± 1.5 to 19.0 ± 0.28%) in the brains of anti-Ly6G treated infected mice (Figure 6C, upper panel). This observation was further confirmed with a significant decrease of Ly6G^+^ neutrophils (Figure 6C, middle panel; 3.0 ± 0.5% to 0.6 ± 0.3%). Further investigation revealed reduced recruitment of CD11b^+^Ly6C^hi^ inflammatory monocytes (Figure 6C, lower panel; 48.0 ± 3.0% to 32.0 ± 1.3%) in the brains of anti-Ly6G treated mice. Moreover, absolute cell numbers of CD45^hi^ CD11b^+^ myeloid cells (*p* < 0.05), Ly6G^+^ neutrophils (*p* < 0.001) and Ly6C^hi^ monocytes (*p* < 0.01) were significantly reduced. On the contrary, numbers of T lymphocytes (CD45^hi^CD11b^−^CD4^+^ and CD45^hi^CD11b^−^CD8^+^) and microglia (CD45^int^CD11b^+^) remained unaltered (Figures 6D,E).

**Figure 7:**
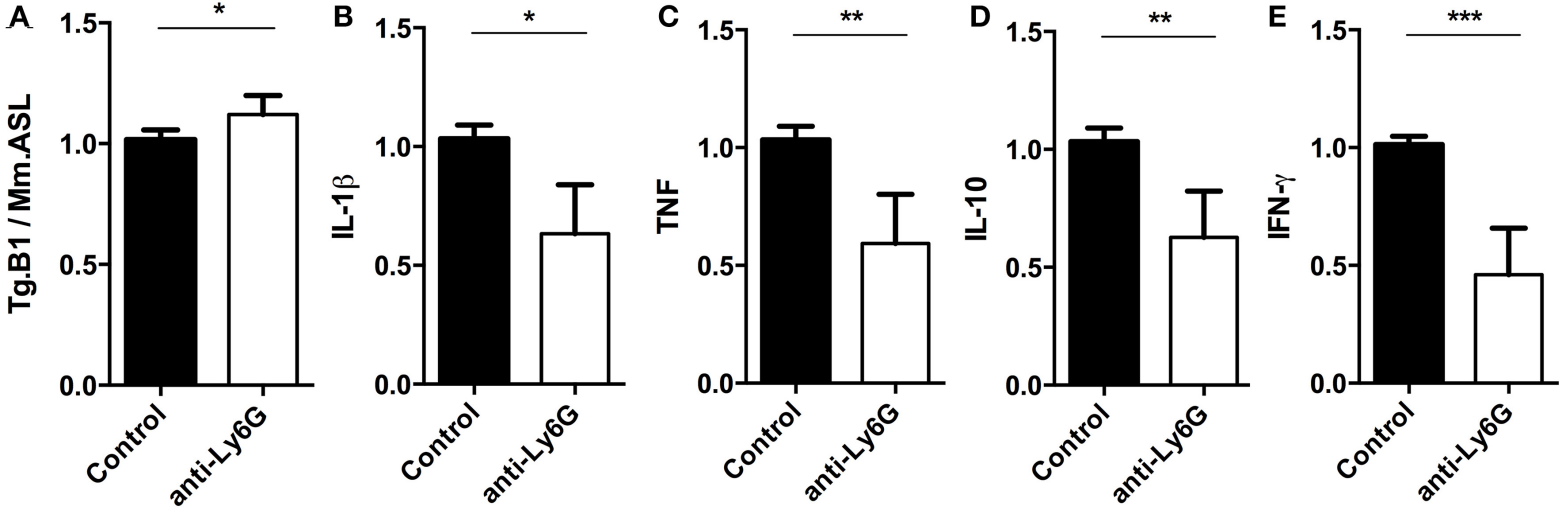
Semi-quantitative RT-PCR analysis of Ly6G^+^ neutrophil granulocytes-depleted mice. Quantitative and Semi-quantitative RT-PCR analyses of pathogen load and cytokine gene expression in brains of *T. gondii*-infected C57BL/6 mice (for 23 days) after treatment with IgG mAb (black bars) or anti-Ly6G (white bars). **(A–E)** Relative expression was calculated by normalization to the expression of the housekeeping genes mouse argininosuccinate lyase and hypoxanthine phosphoribosyltransferase. Resulting data were further normalized on mean values of control groups. Data shown is the representative of 2 individual experiments with 4 mice per group for each experiment. Significant differences (^*^p < 0.05, ^**^p < 0.01, ^***^p < 0.001) were determined using the Mann-Whitney test.

Using q and qRT-PCR we investigated whether the ablation of Ly6G^+^ neutrophil granulocytes affected parasite burden and cytokine gene expression levels in brains of anti-Ly6G treated mice. We found that anti-Ly6G treatment was associated with elevated parasite burden (Figure 7A). Correspondingly, we observed a 40 and 60% reduction of pro-inflammatory TNF and IFN-γ, respectively, as compared to IgG treated controls (Figures 7C,E). Furthermore, IL-10 and IL-1β mRNA levels were reduced in anti-Ly6G treated mice (Figure 7B,D). Together, these results demonstrate that increased parasite burden correlates inversely with the reduced levels of TNF, IFN-γ, IL-1 β, and IL-10 in brains of neutrophil-depleted mice with cerebral toxoplasmosis. Hence, the recruitment of neutrophil granulocytes and their early cytokine production appear to promote inflammatory immune responses restricting pathogen load in brains of *T. gondii*-infected mice.

### Heterogeneity of neutrophil granulocyte subsets in cerebral toxoplasmosis

**Figure 8:**
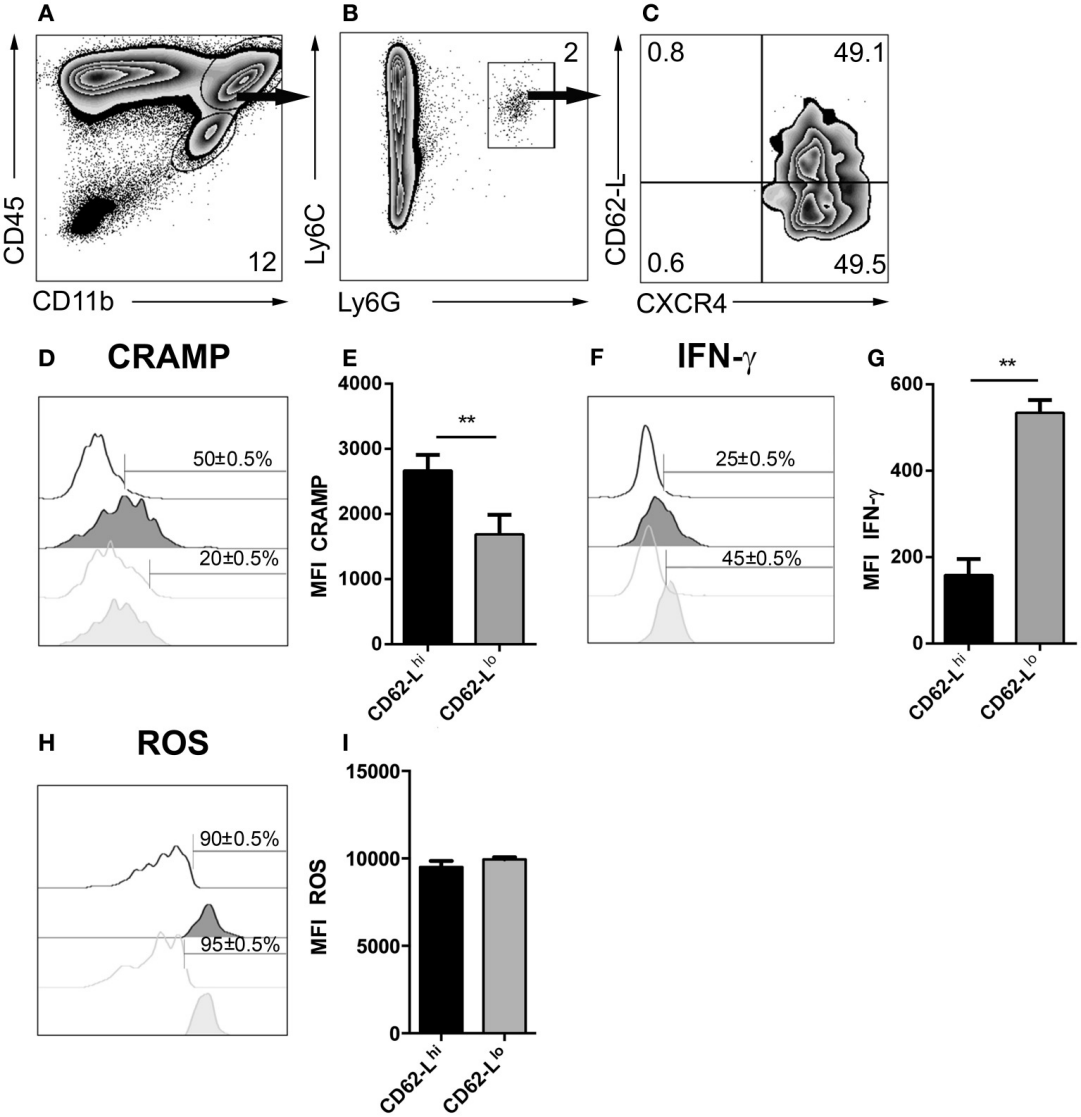
Emergence of neutrophil subsets in the CNS. **(A,B)** Cells isolated from 4 week *T. gondii*-infected mice were gated on CD45^+^CD11b^+^Ly6C^+^Ly6G^+^ neutrophils **(C)**, and their CXCR4 and CD62-L expression was determined. The neutrophil granulocyte subsets CXCR4^+^CD62-L^lo^ and CXCR4^+^CD62-L^hi^ were studied for their CRAMP, IFN-γ and ROS expression. **(D,F,H)** Representative histograms show CRAMP, IFN-γ, and ROS expression of CXCR4^+^CD62-L^hi^ (black filled) and CXCR4^+^CD62-L^lo^ (gray filled) neutrophils as compared to the corresponding isotype control (black or gray, not filled). Bars mark the cells positive for the particular markers. Numbers above the bars represent percentage of cells positive for the marker of the respective population: CXCR4^+^CD62-L^hi^ (black) and CXCR4^+^CD62-L^lo^ (gray). **(E,G,I)** Bar graphs represent the MFI of the respective fluorochrome for a particular marker, MFI ± SD (*n* = 4) (CXCR4^+^CD62-L^hi^ (black) and CXCR4^+^CD62-L^lo^ (gray)). The numbers in the representative contour plots are % of the parent population. Data are representative of 2 independent experiments with *n* = 4 mice per experiment. Significant differences (^**^p < 0.01) in the bar graphs were determined using the Mann-Whitney test.

Depending on the infection model, different subsets of neutrophil granulocytes can be discriminated based on their phenotypic and functional properties (Tsuda et al., 2004; Pillay et al., 2010; Beyrau et al., 2012). However, in the case of cerebral toxoplasmosis, it was largely unclear whether recruited neutrophils represent a heterogeneous or rather homogeneous population in the brain. Therefore, CD45^+^CD11b^+^Ly6C^+^Ly6G^+^ neutrophil granulocytes from 4 weeks *T. gondii*-infected mice were analyzed by flow cytometry (Figures 8A,B). We found that this population contained cells expressing different levels of CD62-L (Figure 8C). Contrary to CD62-L^lo^ neutrophils (20 ± 0.5%), a high percentage of CD62-L^hi^ neutrophils (50 ± 0.5%) expressed CRAMP (Figures 8D,E). On the contrary, only 25 ± 0.5% of CD62-L^hi^ neutrophils secreted IFN-γ while this was the case for 45 ± 0.5% of CD62-L^lo^ neutrophils (Figures 8F,G). However, CD62-L^hi^ and CD62-L^lo^ neutrophil granulocytes produced similar levels of ROS (90 ± 0.5% vs. 95 ± 0.5%) (Figures 8H,I). These data suggest the phenotypic heterogeneity of brain-derived neutrophil granulocytes further defining CD62-L^lo^ neutrophils as a source of protective IFN-γ.

## Discussion

Neutrophil granulocytes are the first immune cells recruited from the periphery to secrete initial effector molecules upon injury and infection. They play a critical role in infection to eliminate pathogens via multiple mechanisms (Zhou et al., 2003; Nathan, 2006; Kolaczkowska and Kubes, 2013). The response of neutrophil granulocytes to parasitic infection is well described in the periphery, but their role within the CNS in chronic infections is not thoroughly defined. One previous study concluded that neutrophil granulocytes are the limiting factor against uncontrolled tachyzoite replication in cerebral toxoplasmosis (Bliss et al., 2001), but this conclusion was based on depletion of Gr1^+^ cells. However, later it turned out that Gr1 expression is not restricted to neutrophil granulocytes but also expressed by monocytes (Daley et al., 2008).

We defined the phenotype and function of Ly6G^+^ neutrophil granulocytes in the course of chronic toxoplasmosis in the CNS. Initially, we observed increased percentages of Ly6G^+^ neutrophils in the blood of *T. gondii*-infected mice. In the chronic stage of the infection, brain resident microglia cells displayed an activated phenotype, and peripheral immune cells including CD11b^−^ lymphoid and CD11b^+^ myeloid cells infiltrated the brain. In line with previous reports, characterization of brain immune cells revealed that recruited CD11b^+^ myeloid cells contained Ly6G^−^ inflammatory monocytes and Ly6G^+^ neutrophil granulocytes (Möhle et al., 2014; Biswas et al., 2015). Despite low absolute numbers of neutrophils in the chronic stage, they form a substantial portion of the myeloid cell compartment in the CNS in the acute stage of the infection. Confocal microscopic analysis identified neutrophil granulocytes adjacent to *T. gondii* tachyzoites in the acute infection, albeit they were distant from cysts in the chronic stage. This observation suggests that the active infective form of the parasite could potentially trigger the host defense and effector functions of neutrophil granulocytes such as generation of ROS or even phagocytosis as it has been shown in acute toxoplasma infection in the periphery (Abdallah et al., 2011).

We measured MHC I and MHC II expression on both microglia and neutrophil granulocytes. This indicates that neutrophils can acquire an APC phenotype in cerebral toxoplasmosis. Neutrophil granulocytes are generally regarded as professional phagocytes responding early to tissue infection and injury. In line with our observations, increasing evidence suggests that neutrophils can also modulate adaptive immune responses by activating CD4^+^ T cells *in vitro* via upregulation of MHC class molecules (Wagner and Hug, 2005; Abdallah et al., 2011). On the contrary, the co-stimulatory molecule CD86 and the phagocytosis-related receptor CD64 (FcγR1) were detected primarily on activated microglia as opposed to neutrophils. The chemokine receptor CXCR2, typically expressed by neutrophil granulocytes, was down-regulated upon entering the brain (Liu et al., 2010). Despite being also expressed on oligodendrocytes and neurons (Liu et al., 2010), where it mediates a wide range of functions, CXCR2 is not present on the activated microglia. Similarly, activated microglia do not express CD62-L which is crucial for leukocyte rolling, transmigration and accumulation at sites of inflammation (Rainer, 2002; Biswas et al., 2015). We identify the existence of two subsets of neutrophils in the infected brain, which can be discriminated based on their differential CD62-L expression (Zenaro et al., 2015). CXCL12, the ligand of CXCR4 is constitutively expressed on endothelial cells in the CNS (McCandless et al., 2006; Wilson et al., 2010). We showed that cerebral toxoplasmosis leads to increased expression of CXCR4 on neutrophil granulocytes. In conclusion, we demonstrate that CNS infiltrating neutrophils display distinct phenotypes in cerebral *T. gondii* infection.

Following surface characterization, we studied the secretion of certain inflammatory mediators by Ly6G^+^ neutrophil granulocytes and activated microglia. The proinflammatory molecules IL-1β and ROS were primarily produced by neutrophils in line with previous studies (Bardoel et al., 2014). Most importantly, we detected that the cytokine IFN-γ, which is the main driving factor of the host immune response against *T. gondii*, was secreted by neutrophil granulocytes. Neutrophils can produce IFN-γ in *Nocardia asteroides*-infected lungs (Ellis and Beaman, 2002) and *Salmonella typhimurium*-infected intestines (Kirby et al., 2002; Sturge et al., 2013). Our observation are in contrast to Sa et al. (2015) who reported that microglia are the main source of myeloid cell-derived IFN-γ in a reactivation model of cerebral toxoplasmosis in RAG^−/−^and IFN-γ^−/−^ mice with a Balb/c background. However, it is important to stress that our results were obtained in WT C57BL/6 mice, which may explain the disparity. Interestingly, our observation is in line with Sturge et al. (2015) who reported that the neutrophils store IFN-γ at the promyelocyte stage in the absence of inflammation. Additionally, they showed that during acute toxoplasmosis neutrophils form an important non-lymphoid source of IFN-production (Sturge et al., 2013). Importantly, we reveal that infiltrating neutrophil granulocytes are an important source of myeloid cell-derived IFN-γ particularly in the early phase of cerebral toxoplasmosis.

The ablation of Ly6G^+^ neutrophils in the periphery resulted in a reduced infiltration to the CNS. The depletion was also associated with a significant reduction of IFN-γ mRNA levels in infected brains. IFN-γ is crucial for survival during *T. gondii* infection (Gazzinelli et al., 1993; Hunter et al., 1994; Gavrilescu et al., 2004; Kim et al., 2012; Gazzinelli and Sher, 2014). Increased parasite loads after anti-Ly6G treatment may have been a direct consequence of the antibody-mediated elimination of IFN-γ-producing neutrophils. Nonetheless, we cannot exclude indirect effects such as those resulting from impaired recruitment of other immune cells. It is important to mention that the neutrophil granulocytes ablation had no effect on the recruitment of CD4^+^ and CD8^+^ T lymphocytes to the brain. This suggests non-significant interaction with the T lymphocytes in terms of recruitment, however T cell activation may have been affected as it not part of the investigation. One study reported that ablation of neutrophils resulted in impaired maturation of resident microglia and brain macrophages into professional APCs, altering the T cell function (Steinbach et al., 2013). In contrary, we did not detect impaired differentiation or activation status of microglia into MHC II expressing professional CD11c^+^ APCs (data not shown).

We previously showed that Ly6C^hi^ monocytes express TNF and IL-10 in the CNS during cerebral toxoplasmosis (Biswas et al., 2015). Thus, the reduction of TNF and IL-10 levels after neutrophil depletion may be due to the reduced recruitment of Ly6C^hi^ monocytes. However, the reduced IFN-γ and IL-1β levels were most likely caused by the complete ablation of Ly6G^+^ neutrophil granulocytes as there was no change in the lymphocyte compartment.

In the current study, based on CXCR4 expression, neutrophil granulocytes displayed certain heterogeneity. Ly6G^+^ neutrophils comprised of CD62-L^hi^CXCR4^+^ and CD62-L^lo^CXCR4^+^ subsets. On one hand the Ly6G^+^CD62-L^hi^CXCR4^+^ subset expressed higher levels of CRAMP and produced lower levels of IFN-γ, but on the other hand Ly6G^+^CD62-L^lo^CXCR4^+^ neutrophil granulocytes expressed lower levels of CRAMP and produced higher levels of IFN-γ. Differential expression of CRAMP and IFN-γ suggest functional heterogeneity of Ly6G^+^ neutrophil granulocytes. One subset Ly6G^+^CD62-L^hi^CXCR4^+^ with high CRAMP expression may promote monocyte recruitment. The Ly6G^+^ neutrophil granulocytes have been described to influence the recruitment of monocytes in injury models (Zhou et al., 2003; Soehnlein et al., 2008a,b). The other subset Ly6G^+^CD62-L^lo^CXCR4^+^ with higher IFN-γ production may establish the inflammatory response against cerebral toxoplasmosis.

Here we report that cerebral toxoplasmosis leads to infiltration of Ly6G^+^ neutrophil granulocytes to the CNS. These neutrophils contribute to IFN-γ production during the early stages of the developing neuroinflammation. Importantly, we identified two distinct Ly6G^+^CXCR4^+^ neutrophil granulocyte subsets based on their CD62-L and CRAMP expression. Moreover, we detected a neutrophil-dependent recruitment of Ly6C^hi^ monocytes to the CNS in chronic *T. gondii* infection. In summary, our findings suggest that neutrophil granulocytes perform important functions to promote host defense and exhibit heterogeneity in experimental cerebral toxoplasmosis.

## Author contributions

ID designed the experiments and supervised the project. AB, TF, HD, NM, MR, AD, UB, performed the experiments. AB, AD, UB, ID, and TS interpreted the data. AB, ID, and TS contributed to manuscript preparation. ID funded the project.

### Conflict of interest statement

The authors declare that the research was conducted in the absence of any commercial or financial relationships that could be construed as a potential conflict of interest.
